# The Potential Benefit of Expedited Development and Approval Programs in Precision Medicine

**DOI:** 10.3390/jpm11010045

**Published:** 2021-01-14

**Authors:** Ariel Kantor, Susanne B. Haga

**Affiliations:** 1 Center for Applied Genomic & Precision Medicine, Duke University School of Medicine, Durham, NC 27708, USA; ariel.kantor@sjc.ox.ac.uk; 2 Nuffield Laboratory of Ophthalmology, Nuffield Department of Clinical Neurosciences & NIHR Oxford Bio-Medical Research Centre, University of Oxford, Oxford OX3 9DU, UK; 3 Headland Strategy Group, 44 Montgomery Str., Suite 300, San Francisco, CA 94104, USA

**Keywords:** regulation, drugs, precision medicine, expedited review, orphan drugs

## Abstract

Background: Increased understanding of the molecular causes of disease has begun to fulfill the promise of precision medicine with the development of targeted drugs, particularly for serious diseases with unmet needs. The drug approval regulatory process is a critical component to the continued growth of precision medicine drugs and devices. To facilitate the development and approval process of drugs for serious unmet needs, four expedited approval programs have been developed in the US: priority review, accelerated approval, fast track, and breakthrough therapy programs. Methods: To determine if expedited approval programs are fulfilling the intended goals, we reviewed drug approvals by the US Food and Drug Administration (FDA) between 2011 and 2017 for new molecular entities (NMEs). Results: From 2011 through 2017, the FDA approved 250 NMEs, ranging from 27 approvals in 2013 to 46 in 2017. The NME approvals spanned 22 different disease classes; almost one-third of all NMEs were for oncology treatments. Conclusions: As these pathways are utilized more, additional legislative changes may be needed to re-align incentives to promote continued development of innovative drugs for serious unmet needs in a safe, efficacious, and affordable manner.

## 1. Introduction

One of the major pillars of the personalized (or precision) medicine revolution is the development of targeted treatments based on increased understanding of the molecular basis of disease. Tremendous progress has been made in defining the molecular etiology and mechanisms of disease through large datasets, advanced genomic technologies, and analytical methods. Moreover, recent advances in basic biomedical sciences and resulting technologies, such as gene editing and adoptive cell transfer, hold great promise for addressing unmet medical needs [1]. One report estimates that the number of approved personalized medicine drugs doubled between 2016 and 2020, jumping from 132 to 286 drugs [2]. Nonetheless, the public health need for continued research and development of new drug and biologic products remains for many diseases as more than 96 percent of rare or orphan diseases still lack effective therapies [3].

The path from initial demonstration of a potential therapeutic effect to the approval of a drug involves many stages, a process that can take up to 15 years with costs exceeding $1.3 billion [4]. In order for research advances to be realized rapidly through the development of new drugs, diagnostics, and devices, the regulatory approval processes in the United States have been amended to facilitate the development of novel, safe, and efficacious compounds in a timely manner. In particular, a series of review pathways have been developed over the years to provide accelerated approval, expedited review, and financial incentives for new drugs that are considered to offer substantial clinical advances or address the most significant unmet needs [5,6]. This has resulted in the development of four pathways with unique benefits: (1) priority review aims for FDA review in 6 months (vs. 10 months for standard review); (2) accelerated approval permits approval based on surrogate endpoints; and (3) fast-track and (4) breakthrough therapy programs, both intended to reduce the duration of clinical trials through more intensive FDA guidance including regular meetings and communication throughout the full development cycle of the drug [7] (Table 1). In addition, the Orphan Drug Act of 1983 provides orphan status to drugs and biologics, which are intended for the safe and effective treatment of rare diseases defined as those that affect fewer than 200,000 people in the US. The orphan drug designation grants financial incentives including a partial tax credit for clinical trial expenditures, waived user fees, and eligibility for seven years of marketing exclusivity.

As the use of accelerated programs increases, it is crucial to understand the nature and overlap in these trends to determine if the programs, as designed, are meeting their goals. Previous analyses have reviewed trends and implications of expedited drug development and approval programs [8,9,10,11], including the impact on reducing clinical development times [12], patient access [13], pricing [14], therapeutic outcomes [15,16], and short and longer-term clinical safety and efficacy [17,18,19].

To further enhance understanding about how well the accelerated programs are performing in meeting their intended goals, we investigated trends in the most innovative drug approvals through analysis of new molecular entities (NMEs), novel drug products marketed for the first time in the United States. In particular, we sought to analyze trends in industry utilization of its expedited drug development and review programs over time and the clinical, economic, and policy implications of these changes on drug development. Although Naci and colleagues have investigated the impact of expedited approvals on valuation, the authors examined the causal effects of these pathways [8], rather than the direct interaction between company size and frequency of expedited review or approval. Our study is the first to broadly consider the impacts of breakthrough therapy designation on other expedited mechanisms.

## 2. Materials and Methods

### 2.1. Data Collection

To investigate trends in the approvals of novel drugs and the impact of the various expedited approval pathways, we reviewed all drug approvals between 2011 and 2017 for small molecule containing NMEs. To identify all NMEs approved in the US during this period and characterize accelerated approval classifications, data were extracted from publicly available FDA databases and review summaries [20,21,22,23]. We also reviewed the Drugs@FDA monthly drug approval reports database, which includes original new drug approvals and biologic license application approvals. To further confirm the completeness of our dataset, we cross-referenced the drug approvals with the FDA Approved Drug Products with Therapeutic Equivalence Evaluations or, ‘Orange Book’ [24]. For each NME, we recorded the submission date, the approval date, the approved indication(s), and whether the compound had been eligible for any special programs or expedited approval pathways. 

To further characterize accelerated approval classifications within NMEs, we reviewed the FDA Center for Drug Evaluation and Research (CDER) Drug and Biologic Accelerated Approvals database [25]. To identify drugs approved through expedited approvals that were subsequently withdrawn, we used historical sources, including published articles, as well as the Federal Register. We also conducted Google searches for the sponsoring companies with key words including “discontinued”, “discontinuation”, “withdrawal”, and “label change”, as well as reviewed the company pipelines of the drug sponsors to cross-check for any drug withdrawals, discontinuations, or label changes.

### 2.2. Coding and Analysis 

To facilitate data analysis, we coded all datapoints. For the clinical indication, we used the Medical Subject Headings (MeSH) disease list to classify each drug into one of 26 different therapeutic categories. For drugs associated with multiple MeSH codes, we examined the earliest available approval documents and other data sources to determine which code most closely corresponded to the clinical indication for the initial approval. For drugs approved for multiple indications, we coded each respective indication with its own disease code.

For each drug approval, if applicable, we documented and coded which of the expedited development and review programs the drug was submitted under: orphan, fast track, accelerated approval, priority review, and breakthrough therapy, as well as whether the drug was first-in-class, a first cycle approval, and/or first in the US. A product may qualify for more than one such program. Drugs were categorized as priority review using the FDA’s annual priority approval reports and the Drugs@FDA database. Drugs were categorized as subpart E (accelerated approval of biological products for serious or life-threatening illnesses) or fast track drugs using the CDER fast track products database. Drugs were categorized as benefitting from accelerated approval using FDA documents including accelerated and restricted approvals under subpart H (drugs), CDER drug and biologic accelerated approvals and the FDA’s annual new drugs summaries. Drugs were characterized as breakthrough designation using the CDER breakthrough therapy designation approvals products database. Drugs were categorized as orphan drugs using the FDA’s monthly drug approval reports database and the FDA’s orphan drug list. A few drugs not listed as orphan drugs in the Drugs@FDA monthly approval reports were listed as having received an orphan designation in the FDA’s orphan drug list; the orphan drug list was utilized if the difference could not otherwise be explained. 

To evaluate the business factors affecting NME drug approvals, including corporate structure and company size, we coded the sponsoring company using the sponsor mailing address included in the drug label or in initial drug approval correspondence. We used a Bloomberg Terminal and Pitchbook to code whether the sponsoring company was private or public, and for publicly traded companies, we quantified company size using the sponsor’s market capitalization as of 31 March 2019. We segmented company size by either small-cap (market capitalization < $2 billion), mid cap (market capitalization $2 billion–5 billion), and large cap (market capitalization > $5 billion). Summary statistics were generated for each datapoint and sub-stratification analysis performed for data with multiple categories. 

## 3. Results

### 3.1. Characterization of 2011–2017 NMEs

**Figure 1:**
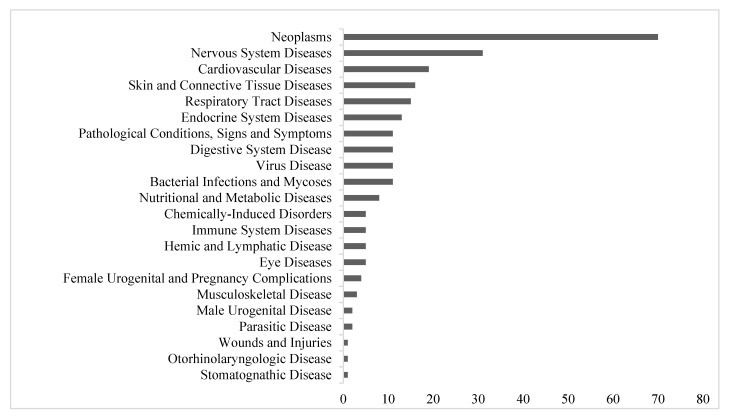
Expedited designations granted to new molecular entities (NMEs) approved the FDA from 2011–2017, divided by therapeutic area.

From 2011 through 2017, the FDA approved 250 NMEs, ranging from 27 approvals in 2013 to 46 in 2017. The NME approvals spanned 22 different disease classes. From the 250 total NME approvals, 4 were indicated in pediatric as well as adult patients, and one was approved solely for pediatric patients. The most common therapeutic areas were oncology (*n* = 70, 28%), nervous system diseases (*n* = 31, 12%), and cardiovascular diseases (*n* = 19, 8%) (Figure 1). First-in-class agents comprised more than one-third of the drugs (*n* = 97, 39%), whereas more than two-thirds were first cycle approvals (*n* = 202, 81%) and almost three-fourths were first approved in the US (*n* = 181, 72%) (data not shown). 

**Figure 2:**
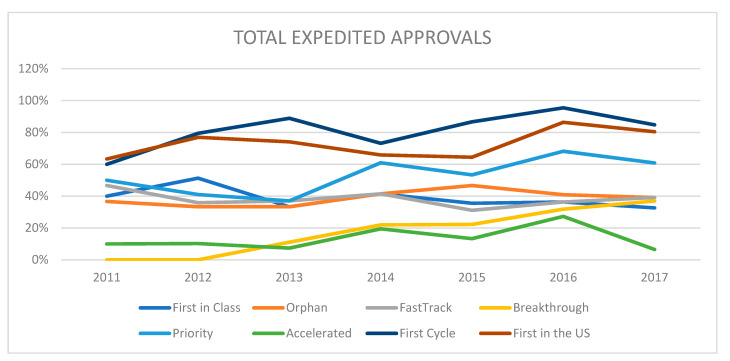
Time trend analysis showing relative percentage of expedited development and FDA review programs granted to each newly approved NME from 2011–2017. Drugs can be associated with more than one program.

Across nearly all therapeutic classes, priority review was the most commonly used programs (*n* = 133), whereas accelerated approval was the least common pathway (*n* = 32) (Figure 2). Oncology was the most prevalent therapeutic category for all four programs—priority review, breakthrough designation, fast track, and accelerated approval. Viral diseases, consisting exclusively of treatments for hepatitis C, received the greatest proportion of expedited program approvals, with 91% priority status, 100% fast track, and 55% breakthrough designation. By contrast, few treatments for skin and connective tissue disorders were observed in any program. Participation rates in the different expedited programs were not always correlated with disease indication. Although 80% of immune system diseases were granted fast track designation, only 40% were granted priority designation and none were granted orphan or breakthrough designation (Figure 1). 

### 3.2. Trends in Expedited Development and FDA Review Programs

Drugs could qualify for more than one program; the average number of expedited development and review programs granted to each newly approved NME varied from a low of 1.21 in 2012 to a high of 2.05 in 2016. When trends in expedited approval pathways were analyzed before the introduction of breakthrough therapy designation (BTD), the five other approval programs maintained similar approval proportions (unpublished data). When further stratified by first-in-class status, the number of NME approvals in the expedited development and review programs assigned for each first-in-class drug reached a maximum of 2.71 in 2014. The lowest number of expedited development programs was also greater for the first-in-class NMEs, with 1.35 in 2012. A greater proportion of breakthrough approvals among first-in-class drugs accounted in part for this difference—while 22% of all NMEs received breakthrough status in 2014, 41% of first-in-class drugs received this designation. Overall, the average number of expedited development and review programs granted over our study timeframe was 1.61 overall and increased to 2.23 among first-in-class drugs. 

### 3.3. Breakthrough Therapy Designation

We observed a steady rise in the adoption of breakthrough therapy designation following the implementation of this pathway by the FDA in 2012. In 2013, the first year of breakthrough status, 11% of total NME approvals (*n* = 3/27) qualified for this designation. Since then, the proportion of breakthrough status approvals has increased steadily, and in 2017, 37% (*n* = 17/46) of NME approvals maintained this designation (Figure 2). 

We further investigated whether these approvals were supplementary in nature or capitalized upon by other expedited approval pathways. The five other expedited programs maintained similar approval proportions over the study timeframe, with a slight downward trend in first-in-class approvals (40% and 51% in 2011 and 2012, compared with 36% and 33% in 2016 and 2017, respectively) and similar proportions for orphan drug, priority status, and accelerated approval. The proportion of first cycle approval and first in the US increased. 

### 3.4. Trends in Expedited Development Programs and First-in-Class Drugs

The frequency of first-in-class NMEs was higher across all accelerated development and review programs other than priority review—56% (*n* = 55/97) of orphan drugs were first-in-class. In comparison, 39% (*n* = 97/250) of the total NMEs received orphan drug designation. About half of fast track designations, breakthrough designations, and accelerated approval designations were also first in class, compared to overall rates of 38%, 18%, and 13%, respectively. For priority review status, 51%were first in class compared to 53% overall. 

### 3.5. Corporate Structure Analysis

A total of 91 different companies based in the US and European Union (EU) sponsored the 250 NMEs approved between 2011 and 2017. Sixty-six (72%) were publicly traded (US or EU) and accounted for 86% of the 250 NMEs approved between 2011 and 2017) (*n* = 215 or an average of 3.25 drug approvals per company), while the 25 privately owned companies accounted for the remaining NME approvals (average 1.4 drug approvals/company). Of the 25 privately owned companies, all are still operational, had post-money valuation of less than $500 million, and, as of March 2019, eight had either merged or were acquired (32%; unpublished data). Of the 66 publicly traded companies, 27 were small-cap, 12 mid-cap, and 27 large cap. The small cap companies sponsored 29 NMEs (average of 1.07 drug approvals/company), while the mid-cap and large-cap companies sponsored 19 (average of 1.58 drug approvals per company) and 167 (average of 6.19 drug approvals/company), respectively.

### 3.6. Trends in Expedited Development Programs and Company Size 

**Figure 3:**
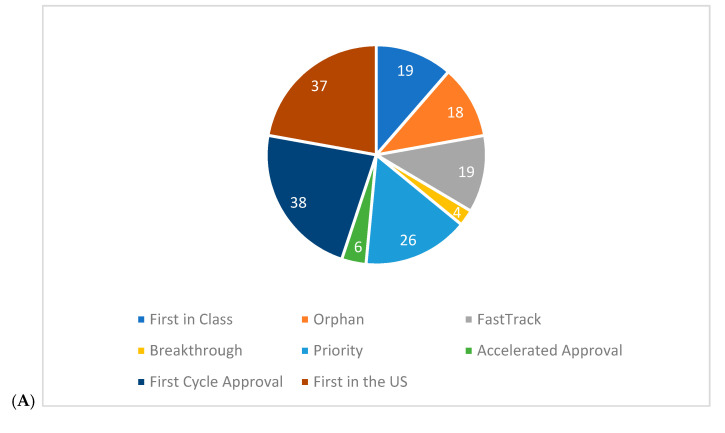

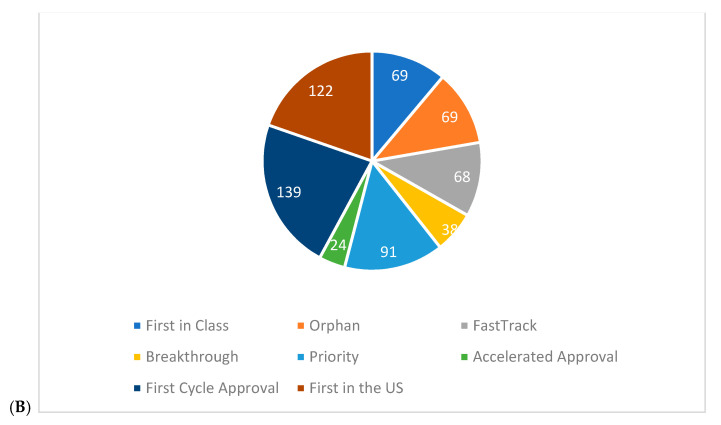
Total number of expedited development and FDA review programs granted to each newly approved NME from 2011–2017, segmented by company market capitalization: (**A**) small/mid cap; (**B**) large cap. Drugs can be associated with more than one program.

To further analyze the relationship between company market capitalization and expedited development programs, we compared small and mid-cap companies combined (39 companies, *n* = 48 NME drugs approved) to large cap companies (27 companies, *n* = 167 NME drugs approved). The proportion of expedited approval pathways and development programs did not vary by company size among priority status, fast track status, accelerated approval, and orphan designation, as well as in first in the US, first cycle, and first in class approval. However, there was a relatively large difference in utility of breakthrough designation status between small and mid-cap and large cap companies (Figure 3). 

### 3.7. Long-Term Safety Follow-Up: Drug Withdrawals, Discontinuations, and Label Changes

Of the 250 NME drug approvals, we identified 10 drug discontinuations, two drug withdrawals, and three drug label changes or safety alerts to reflect unanticipated safety concerns or adverse events. All 10 drug discontinuations were due to business-related factors and not drug safety or efficacy issues. Reasons for discontinuation included scientific advancement and changes in treatment practice leading to reduced product demand, high manufacturing costs coupled with smaller patient populations, and cash flow shortages leading to company decisions to focus on other pipeline candidates. Of the 10 drug discontinuations, there were a total of four first-in-class designations, six fast track designations, two breakthrough designations, and five priority designations. One program was approved through the orphan drug pathway, and none of the drugs benefited from accelerated approval designation. The two drug withdrawals were voluntarily terminated following postmarking reports of serious adverse side effects or other safety or efficacy concerns. Neither drug withdrawal received any expedited approval mechanisms or review pathways. 

## 4. Discussion

Overall, the average number of expedited development and review programs granted over our study timeframe was 1.61 per drug, increasing to 2.23 among first-in-class drugs, with priority review as the most common designation. We observed a steady rise in the adoption of breakthrough therapy designation following its implementation in 2012. The increased proportion of first-in-class drugs supports the benefit of expedited review mechanisms towards the development of new therapies that provide important and novel clinical benefit to patients [26]. These findings are consistent with previous reviews of expedited approval and review pathways showing the increased historical uptick in usage of expedited mechanisms and particularly following the enactment of breakthrough therapy designation [15,18]. 

Although the NME approvals spanned 22 different disease classes, oncology was the most common therapeutic area, encompassing almost one-third of all NMEs. In addition, oncology was the most prevalent therapeutic category across all expedited programs and FDA review mechanisms. Companies seeking oncology approvals often used more than one expediting strategy [10,27,28]. Huang et al. showed that cancer drugs approved through expedited pathways and review mechanisms were associated with faster times to approval despite comparable efficacy to non-expedited drugs [29]. The skew toward oncology treatments will likely continue to increase with greater understanding of genetic drivers and key proteins and development of targeted drugs [30,31,32,33,34,35]. This trend may be exacerbated by the benefit from multiple lines of therapy beyond standard first- or second-line treatment for management of disease progression in oncological indications. Thus, incentivizing development for other untreatable conditions remains a challenge, particularly for small markets that may not allow companies to recoup investments in R&D.

One concern raised about expedited development and approval process of investigational drugs is the potential higher likelihood of safety concerns post-marketing [35,36]; however, the data are conflicting [37,38,39]. Frank et al. showed that drugs approved in the 16 years after the 1992 Prescription Drug User Fee Act (PDUFA) legislation (which led to the enactment of the priority review and accelerated approval pathways) were more likely to receive a new black-box warning or be withdrawn than drugs approved before its passage [40]. The authors suggested that expedited development may have compromised the quality and quantity of evidence submitted to the FDA for review and faster reviews led to overlooked potential risks. Recently, a Lancet Series on Comparative Effectiveness Research addressed some of the ethical and scientific issues that stem from the widespread use of expedited pathways [41,42]. The authors proposed a set of guidelines that may better align incentives for pharmaceutical and device companies and assure timely availability of clinical evidence towards regulatory decision-making. Among their key principles are the need for regulators to be more selective in their use of expedited approval programs based on incomplete clinical data. In our analysis of long-term safety and efficacy, based on drug withdrawals, discontinuations, and label changes, we identified two drug withdrawals (0.8% of total approvals) and three label changes (1.2% of total label changes). These low numbers may be due to the recency of approvals, narrower clinical indications and reduced study population heterogeneity, and more comprehensive pharmacokinetic evaluations. The proportion of drug regulatory follow-up leading to withdrawal or label change was lower among expedited pathways and review mechanisms than among total NME approvals. 

Concerns have also been raised that expedited approval pathways and development programs have resulted in companies profiting excessively [43]. We found that the utility of expedited approval pathways and development programs did not vary between publicly traded and privately-owned companies nor company size (small, mid, and large cap biopharma companies). The only exception to this trend was observed with the breakthrough designation, with four approvals (8% of total approvals) among small cap companies compared to 38 approvals from mid-cap and large cap companies (23% of total approvals). It is possible that the extensive utility of breakthrough status among large companies is reflective of business strategies to reduce R&D costs and the time to profitability. 

Some limitations of this analysis should be noted. In particular, company data are limited and we only reviewed the company listed as the drug sponsor through approval and commercialization. It is possible that a different company may have conducted initial target identification and R&D through earlier clinical phases. In addition, we evaluated the companies as of the date of data collection, March 2019. It is possible that the company had a different corporate structure or market capitalization at the time of drug approval. In particular, since we only evaluated companies that were granted NME drug approvals, the financial data may show a higher valuation than is reflective of traditional biopharma players in the space. 

In conclusion, our analysis shows that the majority of newly approved NMEs are associated with at least one expedited approval or FDA review pathway. With increasing identification of new of molecular drug targets, we anticipate continued growth in breakthrough therapy designation, which we believe will be chiefly pursued by large-cap biopharma companies. Special approval mechanisms address a clear need to rapidly develop new and more effective drugs to address pressing medical needs. As these pathways become more commonly used, future revisions will likely be needed to re-align incentives and ensure continued development of innovative drugs that address serious unmet needs in a safe, efficacious, and affordable manner. 

## Figures and Tables

**Table 1:** Summary of FDA Expedited Development and Review Programs.

	Orphan Drug	Fast Track	Priority Review	Accelerated Approval	Breakthrough Therapy
Year instituted	1983	1988	1992	1992	2012
Eligibility	Treats disease occurring in <200,000 people per year in United States	Drugs intended to treat serious of life-threatening conditions Data demonstrate potential to address unmet clinical need	Drugs that offer major advances in treatment for conditions with no existing adequate treatment Priority review voucher	Drugs that fill unmet need for serious conditions	Drugs intended to treat serious or life-threatening conditions Clinical data suggest more effective than existing therapies
Change clinical trial progression?	No	Yes; can approve after single phase 2 study	No	Yes; can approve on basis of surrogate endpoint reasonably likely to predict patient benefit	No
Phase during which it exerts most direct effect	Drug development	Drug development and FDA review	FDA review	Drug development and FDA review	Drug development and FDA review
Benefit	7-year marketing exclusivity Tax credits Office of Orphan Product Development (OOPD) assistance during the development process	Actions to expedite development and review Rolling review	Expedited NDA review (6 months vs. standard 10 months standard review)	Approval based on surrogate endpoints	Intensive guidance from FDA Rolling review Other actions to expedite review Organizational commitment with senior managers
